# Comparing Sampling Strategies for Tackling Imbalanced Data in Human Activity Recognition

**DOI:** 10.3390/s22041373

**Published:** 2022-02-11

**Authors:** Fayez Alharbi, Lahcen Ouarbya, Jamie A Ward

**Affiliations:** 1 Computer Sciences and Information Technology College, Majmaah University, Al Majmaah 15341, Saudi Arabia; 2 Department of Computing, Goldsmiths, University of London, London SE14 6NW, UK; l.ouarbya@gold.ac.uk (L.O.); j.ward@gold.ac.uk (J.A.W.)

**Keywords:** activity recognition, wearable sensors, imbalanced activities, sampling methods

## Abstract

Human activity recognition (HAR) using wearable sensors is an increasingly active research topic in machine learning, aided in part by the ready availability of detailed motion capture data from smartphones, fitness trackers, and smartwatches. The goal of HAR is to use such devices to assist users in their daily lives in application areas such as healthcare, physical therapy, and fitness. One of the main challenges for HAR, particularly when using supervised learning methods, is obtaining balanced data for algorithm optimisation and testing. As people perform some activities more than others (e.g., walk more than run), HAR datasets are typically imbalanced. The lack of dataset representation from minority classes hinders the ability of HAR classifiers to sufficiently capture new instances of those activities. We introduce three novel hybrid sampling strategies to generate more diverse synthetic samples to overcome the class imbalance problem. The first strategy, which we call the *distance-based method* (DBM), combines Synthetic Minority Oversampling Techniques (SMOTE) with Random_SMOTE, both of which are built around the k-nearest neighbors (KNN). The second technique, referred to as the noise detection-based method (NDBM), combines SMOTE Tomek links (SMOTE_Tomeklinks) and the modified synthetic minority oversampling technique (MSMOTE). The third approach, which we call the cluster-based method (CBM), combines Cluster-Based Synthetic Oversampling (CBSO) and Proximity Weighted Synthetic Oversampling Technique (ProWSyn). We compare the performance of the proposed hybrid methods to the individual constituent methods and baseline using accelerometer data from three commonly used benchmark datasets. We show that DBM, NDBM, and CBM reduce the impact of class imbalance and enhance *F*1 scores by a range of 9–20 percentage point compared to their constituent sampling methods. CBM performs significantly better than the others under a Friedman test, however, DBM has lower computational requirements.

## 1. Introduction

Human activity recognition (HAR) using body-worn or wearable sensors is an active research topic in mobile and ubiquitous computing [1]. Activity recognition is a useful tool because it provides information on an individual’s behaviour that enables computing systems not only to monitor but also to analyse and assist with a range of day-to-day tasks [2,3].

Most HAR studies adopt a supervised learning approach [4]. Supervised learning typically requires immense amounts of labelled sensor data in order to train [2]. For such models to work well, the data are ideally recorded from a variety of real-word situations. Additionally, a diversity of sensor modalities and placements can help improve recognition performance [5,6].

Sensor data obtained from real-life settings is typically of poor quality (noisy) and frequently has missing data [7]. These issues arise due to factors such as bad or faulty placement of sensors, or sensor malfunctioning [8]. Similarly, sensor data may often be highly imbalanced due to significant individual variations, with limited labels for certain activities [9]. Further barriers to obtaining sufficient quantities of real-world data include the prohibitive cost of devices, issues related to privacy, or a desire to reduce battery consumption [10]. Sensor data from certain activities can be difficult to obtain because of the rare but critical nature of those activities, such as falls in the elderly [2] or heart failure [11].

For HAR to succeed as a viable technique, there is an urgent need for new approaches at making up for this shortfall in critical and underrepresented real-world data [2,12]. An important part of this is solving the class imbalance problem [13]. Imbalance can occur in both the between-class distribution and as within-class imbalance [14]. Between-class imbalance occurs, for example, when some activities are performed less often than others [15]. As a result, the sample sizes for these activities are smaller, so a supervised model might not have enough data to learn adequately. The related concept of within-class imbalance occurs when the same activity is performed in different ways by the same individual, yet there are insufficient examples from some of these for the model to generalise adequately [12].

There are usually two methods to solve class imbalance: data level (sampling) methods and algorithm level methods [16]. The data level approach involves changing a training set’s class distribution by resampling. This might mean oversampling the minority classes, undersampling the majority classes, or a combination of both [17]. The algorithm level approach involves adjusting existing learning algorithms to focus more on the minority classes [16]. In this work we use the data level approach, which is less complex to configure and can be integrated with any learning algorithm.

The main contributions of the work are the following.

- We evaluate six sampling methods (SMOTE, Random_SMOTE, SMOTE_Tomeklinks, MSMOTE, CBSO, and ProWSyn) as solutions to the class imbalance problem across three commonly used datasets.
- We introduce three novel hybrid sampling approaches and show how these build on and improve upon their constituent methods. These are (1) DBM, a distance-based method that combines SMOTE and Random_SMOTE, (2) NDBM, a noise detection-based method that combines SMOTE_Tomeklinks and MSMOTE, and (3) CBM, a cluster-based method that combines CBSO and ProWSyn.
- We compare how useful the sampling methods are to improve the learning from imbalanced human activity data using both shallow and deep machine learning algorithms. Specifically, we test KNN, Logistic regression (LR), Random Forest (RF) and Support Vector Machine (SVM), and a Multilayer perceptron (MLP) [18,19]. We show that the sampling methods are only useful to improve the performance of the MLP compared to the other classifiers for imbalanced human activity data.

The remainder of the paper is organised as follows. Section 2, shows some of the existing work on class imbalance problem and techniques to deal with it in HAR. Section 3 provides background on the sampling methods used. Section 3.4 introduces the proposed method and Section 4 describes the datasets. Section 5 describes the data analysis and experimental setup. Section 6 introduces the experimental results, and Section 7 discusses the findings from these.

## 2. Related Work

Several authors have highlighted the importance of the class imbalance problem in HAR [2,12,20,21]. Ni et al. introduced a HAR system based on using the stacked denoising autoencoder (SDAE) to recognise static and dynamic ambulatory activities, such as standing and running, using accelerometers and gyroscopes [22]. The performance of their model dropped as the class (activity) distribution of samples became unbalanced. They used sampling techniques such as SMOTE and random undersampling to tackle the problem. Based on their experimental result, the sampling techniques were more successful than random undersampling at successfully treating imbalance and improving recognition performance. This is because the indiscriminate nature of the undersampling approach can lead to useful data being discarded.

Despite the promising results, Ni et al’s. work is limited in that it oversamples the entire dataset. Implementing oversampling before splitting a dataset into different train and test partitions can result in information leakage from the original test data to the newly produced training data and this can then lead to overly optimistic classification performance [23]. In other words, the learning algorithm’s performance might be less about its ability to generalise to the test data appropriately, than it is an indication of similar patterns in both train and test data due to information leakage. In the current work we avoid the information leakage problem by sampling exclusively on the training set.

Chen et al. [20] used data from accelerometers and gyroscopes to recognise activities such as walking, jogging, and jumping—again using an imbalanced dataset. They reported that the classifier always showed a good performance in recognising the majority class, whereas its performance was inadequate for the minority classes. Again, SMOTE was used to increase the count of underrepresented activities, leading to improved overall performance. One limitation of this work was that it only evaluated a single oversampling method. In contrast, our work evaluates a combination of different methods.

Inspired by the concept of data fusion, we introduce three hybrid sampling methods—DBM, NDBM, and CBM—which combine the outputs from different sampling methods. Fusion of diverse data sources and sensor modalities is a widely explored approach for improving recognition performance in HAR (e.g., [5,18,24,25]). Similarly, fusion of multiple, diverse, weak learners to produce a strong ensemble is a well-studied and effective approach in machine learning [26]. We hypothesise that by combining outputs from different sampling strategies we diversify the synthetic data and in turn improve the generalization ability of our learning models.

## 3. Sampling Methods

The underlying sampling methods used in this work can be categorised into three types: distance-based, noise detection-based, and cluster-based.

### 3.1. Distance-Based

SMOTE and Random_SMOTE both use distance-based algorithms to oversample the training data. SMOTE [27] takes an instance of the minority class *x* from the training set, and then computes its *K* nearest neighbours, identified as the shortest Euclidean distances between itself and other instances of the same class. To produce a synthetic sample, $x_{new}$, SMOTE randomly selects the *K* nearest neighbours from the minority class, e.g., $x_{k}$ for the *k*th nearest neighbour, and computes the difference $x_{k}-x$. The new synthetic sample, $x_{new}$, is computed by multiplying this difference by a random number between 0 and 1 using Equation (1). The new synthetic instance $x_{new}$ will lie along the line between *x* and $x_{k}$:

(1) $$x_{new}=x+||x-x_{k}\left|\right|\times rand\;\left(0,1\right)$$

Unfortunately, the linear design of SMOTE can lead to overfitting. Random_SMOTE [28] tackles this by opening up a much wider region for oversampling. For each minority instance, *x*, two minority samples $x_{1}$ and $x_{2}$ (with $x_{1,2}\neq x$) are randomly selected. A temporary synthetic sample $x_{tmp}$ is then generated along the line between $x_{1}$ and $x_{2}$, as shown in Equation (2):

(2) $$x_{tmp}=x_{1}+\left|\right|x_{2}-x_{1}\left|\right|\times rand\;\left(0,1\right)$$

The final synthetic sample $x_{new}$ is then created along the line between $x_{tmp}$ and the original sample *x* using Equation (3):

(3) $$x_{new}=x+\left|\right|x_{tmp}-x\left|\right|\times rand\;\left(0,1\right)$$

### 3.2. Noise Detection-Based

Real world data contain noise from a variety of sources that can lead to poor recognition performance [29,30]. Frenay et al. [31] indicated that class noise (also known as label noise) is one of the most harmful noises in machine learning. This kind of noise can occur, for example, if a minority class sample is incorrectly labelled with a majority class label [16]. The SMOTE-Tomek Link [32] and modified synthetic minority oversampling technique (MSMOTE) algorithms are specifically designed to detect this kind of noise in order to minimize the risk of creating noisy synthetic samples [33].

SMOTE-Tomek Links oversamples using SMOTE on top of a Tomek link data cleaning step [32]. Tomek link works as follows: Consider two samples $x_{a}$ and $x_{b}$ belonging to different classes, where $d(x_{a},x_{b})$ is the Euclidean distance between $x_{a}$ and $x_{b}$. A Tomek link is identified as an $\left(x_{a}x_{b}\right)$ pair if there is no sample *z* that meets the following conditions: $d\left(x_{a},z\right)<d\left(x_{a},x_{b}\right)$ or $d\left(x_{b},z\right)<d\left(x_{a},x_{b}\right)$. That is, $x_{a}$ and $x_{b}$ are each other’s nearest neighbours [34]. Tomek links are therefore likely to be comprised of either boundary samples or noisy samples [30,35,36]. SMOTE-Tomek Links generates synthetic data in two steps [32]. First, the original minority training data are oversampled using SMOTE. Second, Tomek links are identified in the training data and removed to rebalance the data set.

MSMOTE is an improved version of SMOTE which first uses KNN to assign minority samples into three types: safe, border, and noise [33]. If a minority labelled sample is the same as the labels of its *k* near neighbours, then the sample is defined as ‘safe’. If the labels are all different, then the sample is identified as ‘noise’. Finally, if the sample is neither safe nor noise, it is classed as a ‘border’ sample. The second step of MSMOTE uses SMOTE to generate new samples. However, the random selection of neighbours is different depending on whether the sample is safe, border, or noise. For safe samples, MSMOTE will randomly choose the *K* nearest neighbours. For border samples, the algorithm only selects the nearest neighbour (i.e., K=1). Noise samples are simply disregarded.

### 3.3. Cluster-Based

The cluster-based sampling methods include Cluster-Based Synthetic Oversampling (CBSO) and Proximity Weighted Synthetic Oversampling Technique (ProWSyn).

CBSO integrate clustering and SMOTE-it uses agglomerative clustering to first cluster minority samples with the aim of identifying those minority samples which are close to the majority samples border [37]. CBSO produces samples only in the neighbourhood of minority samples that are close to majority neighbours using SMOTE. For instance, in order to produce a new sample, CBSO will select a sample *x* from the minority class and randomly choose a minority sample $x_{k}$ from *x*’s cluster (using SMOTE Equation (1)), to produce a new sample.

ProWSyn is another cluster-based sampling method [38]. This algorithm computes the distance between minority class samples and majority class samples in order to assign greater weights to the minority samples. These weights are then used to assign greater significance to the minority samples during learning. ProWSyn operates in two steps: The first step splits the minority data into partitions (*P*) according to their distance from the class boundary. ProWSyn assigns a proximity level (*L*) to each partition. The level increases with distance from the boundary. When minority class samples are assigned to lower proximity levels, then they are considered more important for learning because they are close to the boundary. However, in cases where they are assigned higher proximity levels they are considered less important [38].

### 3.4. Proposed Hybrid Methods

**Figure 1:**
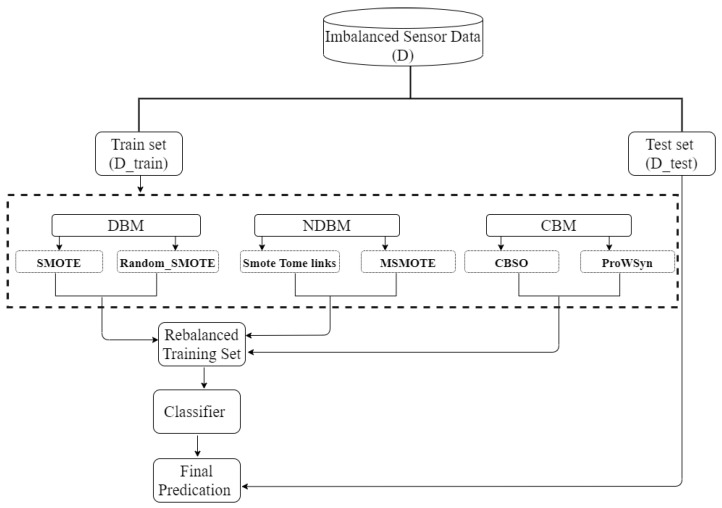
Overview of the process used for splitting, oversampling, and evaluating the data.

All three proposed hybrid approaches, DBM, NDBM, and CBM, concatenate synthesized training data obtained from the constituent sampling methods. DBM combines SMOTE and Random_SMOTE. NDBM combines SMOTE_Tomeklinks and MSMOTE. Finally, CBM combines CBSO and ProWSyn. The three methods are evaluated as shown in Figure 1. Taking *D* as the original dataset, we first split *D* into $D_{train}$ and $D_{test}$. $D_{train}$ is then oversampled using the constituent methods. For more clarification, lets refer to the $D_{train}$ as *d* and, for instance, if *DBM* is used to oversample *d*, it will be:

(4) $$d_{DBM}=SMOTE\left(D_{train},\alpha \right)+Random\text{\_}SMOTE\left(D_{train},\alpha \right)$$

where α is the oversampling ratio. In case of using NDBM, it is denoted as:

(5) $$d_{NDBM}=SMOTE\text{\_}TomekLinks\left(D_{train},\alpha \right)+MSMOTE\left(D_{train},\alpha \right)$$

For the CBM, it is referred to as:

(6) $$d_{CBM}=ProWSyn\left(D_{train},\alpha \right)+CBSO\left(D_{train},\alpha \right)$$

We then concatenate the synthesized data to increase the size of $D_{train}$. The oversampled $D_{train}$ is used to train a classifier, which is then evaluated on the left-aside $D_{test}$.

## 4. Datasets

We use three datasets that are widely used by HAR researchers: Opportunity [39], Physical Activity Monitoring (PAMAP2) [40], and Activities of Daily Living (ADL) [41]. Each of these comprise many individuals performing different types of human activity, including ambulation and daily living activities [42]. The ambulation activities are typically performed over a longer period of time, which comes in two difference forms: static (less repetitive) such as standing, or dynamic (more repetitive), for example, running. Shoaib et al. [43] describe these activities as ‘simple’ because they might be easily identified using a wrist-worn accelerometer placed at an individual wrist. Daily activities might consist of hand gestures such as waving hands or hand-to-mouth gestures (HMG), for example, eating or drinking [44]. Daily activities are not as repetitive as ambulatory dynamic activities, and these daily activities often are concurrent with each other due to their similar gestures such as eating, drinking, and brushing teeth [45]. Such activities are referred to as ‘complex’ because they are more challenging to identify using a single accelerometer compared to simple activities [43].

### 4.1. Opportunity

**Figure 2:**
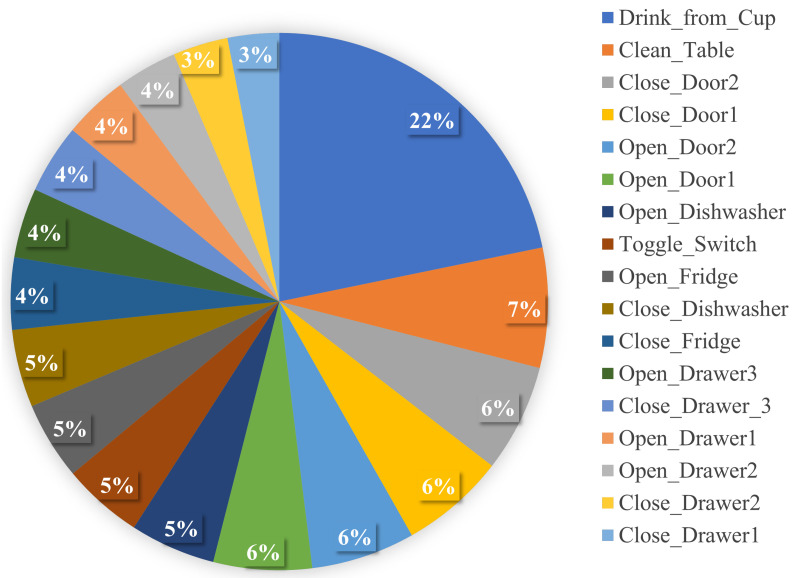
Activity distribution of the Opportunity dataset.

The Opportunity dataset was collected from 72 sensors, with different types of sensors integrated into the environment, objects and worn on participants’ bodies [39]. Four participants performed daily living scenarios in a simulated kitchen environment. The dataset included around 6 h of recordings and was sampled at 30 Hz. The activities were annotated on two different levels: locomotion and gesture. For example, *cleaning up* and *open* door were labelled as gestures, with *sitting* and *lying* making up the locomotion subset. Here we focus solely on gesture activities. Figure 2 shows 17 activities categorised as gestures, including *Open Door1, Open Door2, Close Door1, Close Door2, Open Fridge, Close Fridge, Open Dishwasher, Close Dishwasher, Open Drawer1, Close Drawer1, Open Drawer2, Close Drawer2, Open Drawer3, Close Drawer3, Clean Table, Drink from Cup*, and *Toggle Switch*. The dataset contains several on-body and object sensors, but in this paper we use only the accelerometer in the lower right arm-worn inertial measurement unit (IMU).

### 4.2. PAMAP2

**Figure 3:**
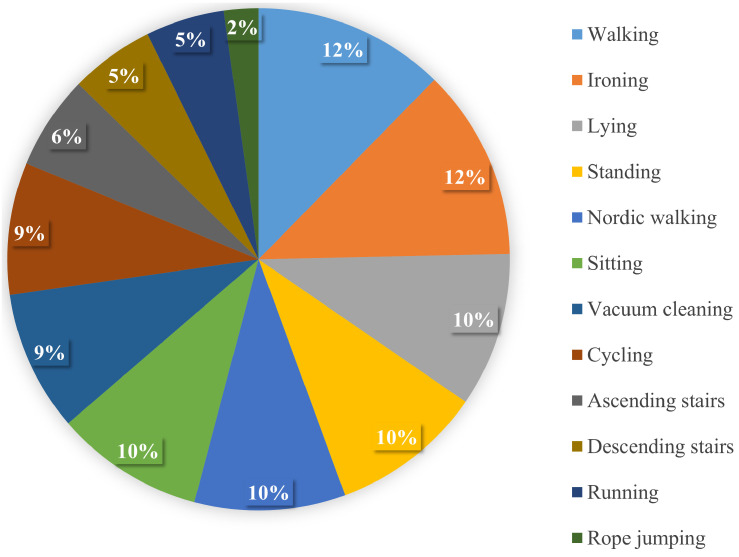
Activity distribution of the PAMAP2 dataset.

The Physical Activity Monitoring dataset (PAMAP2) was collected from 9 participants who performed 12 activities for over 10 h and it was sampled at 100 Hz. Data were recorded by using IMUs placed on the hand, chest, and ankle [40]. Here we use only the accelerometer sensor of the hand-worn IMU. Figure 3 shows the activity distribution, and it can be seen that the dataset is imbalanced. It contains both simple and sporting activities such as walking, running, cycling, Nordic walking, and rope jumping. It also includes posture activities such as lying, sitting, and standing. Activities of daily living (ascending stairs, descending stairs), and households activities such as vacuum cleaning and ironing are also included.

### 4.3. ADL

**Figure 4:**
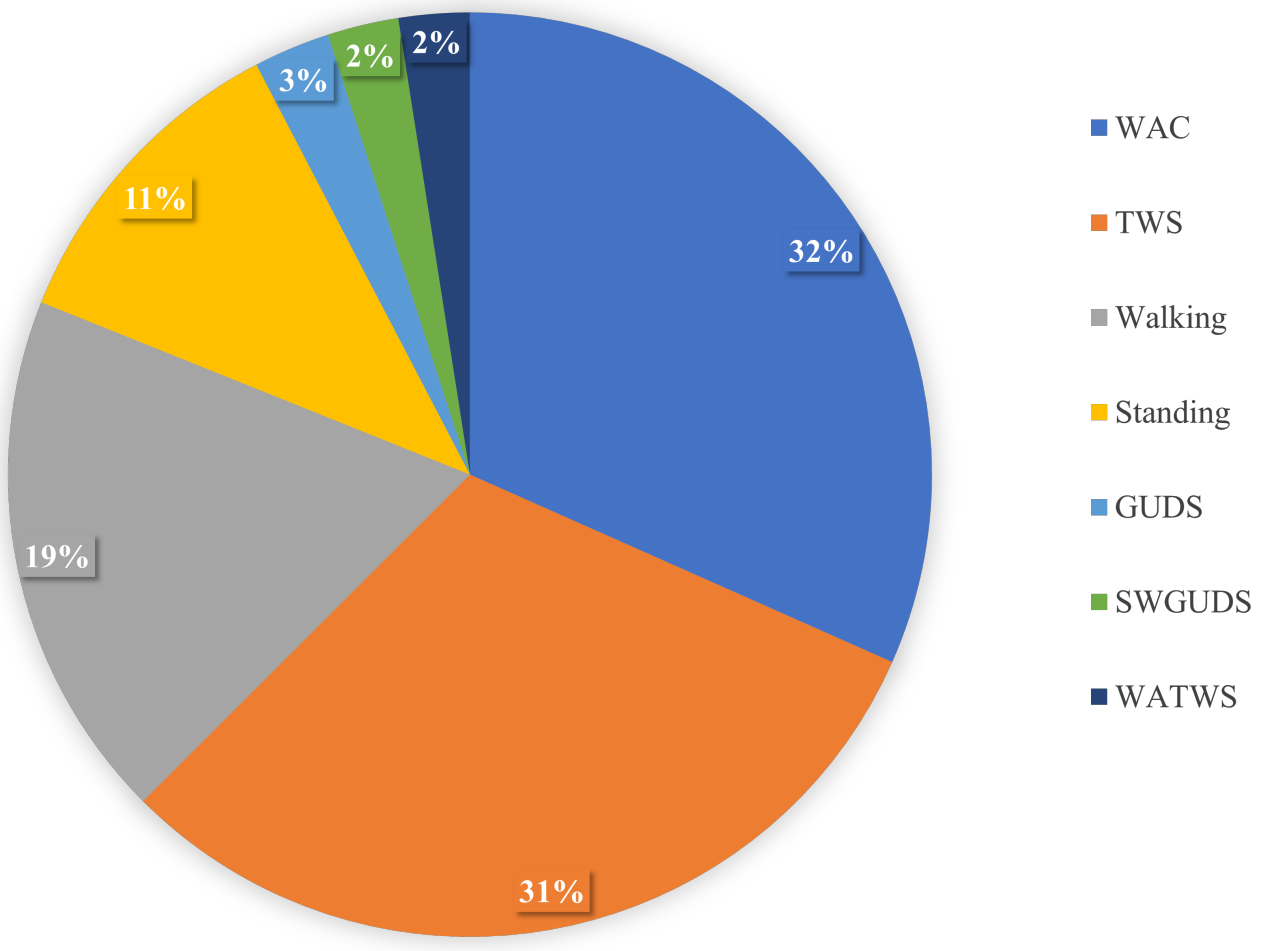
Activity distribution of the ADL dataset.

The Activities of Daily Living (ADL) dataset is a public dataset collected using a single chest-worn wearable accelerometer on 15 participants [41]. The sampling rate of the accelerometer was 52 Hz. The participants performed seven daily living activities. The activities include *Working at Computer* (*WAC*), *Standing Up*, *Walking and Going Up*/*Downstairs* (*SWGUDS*), *Standing, Walking*, *Going Up*/*Downstairs* (*GUDS*), *Walking and Talking with Someone* (*WATWS*), and *Talking while Standing* (*TWS*). Figure 4 shows the activities distribution of the ADL dataset which indicates that the dataset is imbalanced.

## 5. Data Analysis

### 5.1. Data Preprocessing

We explore how our proposed sampling methods might enhance a human activity model’s performance in a real-life scenario where only a single 3-axis accelerometer is available. Although recognition performance is typically better when multiple sensors are used, in many scenarios access to multiple sensors is limited (e.g., in a single wrist-worn device) [10].

As a pre-processing step, we first calculate the Euclidean norm ($\sqrt{x^{2}+y^{2}+z^{2}}$) of each 3-axis sensor to ensure the data are invariant to shifting orientation of the sensors [46]. We then apply a non-overlapping sliding window to segment the data [24]. Table 1 provides more details such as the number of subjects, sampling rate, the window size, and sensor position we use.

We extract six time-domain features including mean, standard deviation, minimum, maximum, median, and range. The selected features are highlighted further in Table 2. These features are efficient as well as fast to compute [18].

### 5.2. Parameters Setting

All of the evaluations in this work were carried out using a collection of shallow learning methods—specifically, SVM, LR, kNN, and RF—as well as a deep learning method based on MLP. The parameters for SVM, LR, and KNN were found using grid search (see Appendix C for details). For RF and MLP, we use the default settings provided by the Python implementation [47]. The MLP architecture that was used in the experiments is presented in Table 3.

Note that for brevity we include only the full results related to the overall best-performing classifier, MLP. The main findings using the remaining classifiers were broadly in agreement (as will be shown in Section 6.7). The full results for the remaining four classifiers are provided in Appendix B.

We also use the default settings on the Python implementation of our sampling algorithms—SMOTE, Random_SMOTE, SMOTE_TomekLinks, MSMOTE, CBSO, and ProWSyn. Number of neighbours and the number of samples to generate are common parameters among all sampling methods. In addition, other sampling methods use specific parameters, for example, ProWSyn utilizes number of levels. Ref. [37] provides more details about these parameters.

The percentage of samples to be created by a sampling method was set to 100%, which means that the number of minority samples in the training set will be equal to the number of majority samples in the training set after sampling.

### 5.3. Evaluation Method

Performance is measured using weighted *F*1 *score*, *recall*, and *precision* [12]. *Precision* records the proportion of class predictions that are correct, whereas *Recall* records the proportion of actual class samples that are correct [48]. The weighted *F*1 *score* used here weighs classes based on their sample proportion and is calculated as (e.g., [1]):

(7) $$\begin{array}{c} F1\;score=\sum_{i}2*w_{i}\frac{\times Precision_{i}\times Recall_{i}}{Precision_{i}+Recall_{i}} \end{array}$$

Here, *i* corresponds to the class. $w_{i}=n_{i}/N$ corresponds to the proportion of class *i* and $n_{i}$ is the number of samples of the class *i*. *N* is the total number of samples.

Pirttikangas et al. [49] suggested to combine all the data from different subject into one dataset. They augured this was because of the individual variation in body worn acceleration which is often dominates by strong commonalities among individuals in activity patterns.

Consequently, we followed Pirttikangas et al.’s [49] suggestion in this work and used 3-fold cross-validation to train the parameters in our analyses. We did not use more than 3 folds as most of the activities have a very low number of samples in some datasets. As an additional measure of reliability, our evaluations are all repeated 30 times using different random selections of data. The final results are presented as the mean (and standard deviation) of the *F*1 *score* over these repetitions. In the future we aim to use a leave-one-subject-out approach.

ANOVA and Friedman statistical tests were performed to discover whether there are significant differences in performance between the sampling methods across the five classifiers [50,51].

## 6. Results

Here we present the final results of each of the sampling methods and our hybrid methods vs the baseline (no resampling) case for each of the three datasets. We also present an evaluation of the power considerations for each method.

### 6.1. Distance-Based Method (DBM)

Table 4 shows the main results for the MLP classifier using DBM versus its constituent methods, SMOTE, and Random_SMOTE. The first thing to notice is a universal improvement when sampling is used compared to the baseline.

On the ADL dataset, the DBM *F*1 score is 92.59%, a 5.39 percentage point (pp) improvement over baseline, a 0.35% improvement over SMOTE, and a 1.52 pp improvement over Random_SMOTE. On Opportunity, DBM’s *F*1 score is low (48.49%), however, this is a large 19.64 pp improvement on the baseline, and a 5 pp improvement over both constituent sampling methods. On PAMAP2, DBM *F*1 score is 80.15%, which is an 8.3 pp improvement on the baseline, and 5 pp on both constituent methods.

### 6.2. Noise Detection-Based Method (NDBM)

Table 5 demonstrates the MLP classifier performance of baseline, NDBM, SMOTE_TomekLinks, and MSMOTE across all datasets. Again, a large improvement is evident for all datasets when using sampling versus baseline.

On the ADL dataset, the NDBM *F*1 score is 93.7%, a 5.39 pp improvement over the baseline, and between 1–2 pp improvement over the constituent sampling methods. On the Opportunity dataset, NDBM performance is low (with *F*1 at 46.95%)—however, this is a dramatic 18.1 pp improvement over the baseline case. On the PAMAP2 dataset, the NDBM *F*1 score is 79.43%, a 7.58 pp improvement on the baseline, and 5 pp improvement over both constituent methods.

### 6.3. Cluster-Based Method (CBM)

Table 6 demonstrates the MLP classifier performance of baseline, CBM, CBSO, and ProWSyn across all datasets. Again, a clear improvement is evident for all datasets when using sampling versus baseline.

On the ADL dataset, the CBM *F*1 score is 92.96%, a 5.76 pp improvement over the baseline, and a 1.8–1.4 pp improvement over the constituent sampling methods. On the Opportunity dataset, CBM performance is low (with *F*1 at 48.87%)—however, this is a dramatic 20.02 pp improvement over the baseline case. On the PAMAP2 dataset, the CBM *F*1 score is 81.15%, a 9.13 pp improvement over the baseline, and a 5.29 pp and 6.56 pp improvement over CBSO and ProWSync, respectively.

### 6.4. Comparing the Performance of the Proposed Sampling Approaches DBM, NDBM, and CBM

Table 7 combines the headline results from our proposed hybrid methods. On the ADL dataset, CBM outperforms the others, with an *F*1 score of 92.96%. On Opportunity, CBM (48.87% *F*1) narrowly outperforms DBM (48.49% *F*1) and NDBM (46.95% *F*1). Similarly, on PAMAP2, CBM (80.98% *F*1) outperforms DBM (80.15% *F*1) and NDBM (79.43% *F*1). The standard deviation across recognition scores for all evaluations is low throughout, with the maximum deviation being no more than 0.087.

### 6.5. Results for Minority Activities

**Figure 5:**
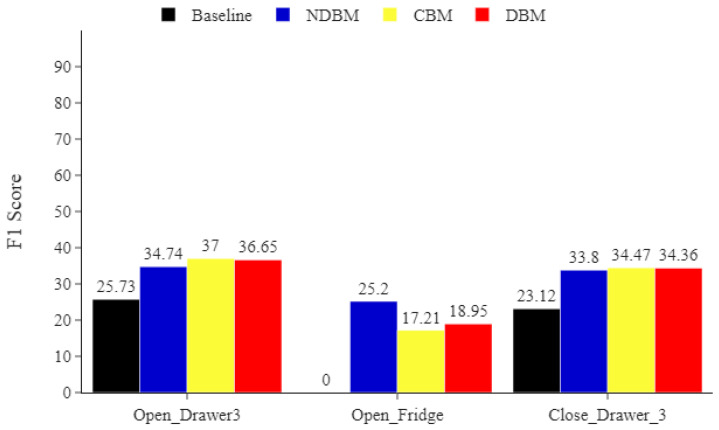
Opportunity minority classes. Comparing the impact of DBM, NDBM, and CBM on activity recognition performance, using MLP for the most underrepresented activities *Open_Fridge*, *Open_Drawer3*, and *Close_Drawer3*. The reported means of *F*1 scores are obtained from 30 repetitions. The *F*1 score is in %.

For the Opportunity dataset, multiple activities were underrepresented, such as *Open_Fridge*, *Open_Drawer3*, and *Close_Drawer3*. Figure 5 indicates that the proposed DBM, NDBM, and CBM improve the *F*1 score of the MLP in recognising the underrepresented activities. Figure 5 also shows that without applying the sampling methods (baseline), the MLP classifier could not identify the *Open_Fridge* activity. By applying the proposed sampling methods, the MLP’s ability to recognise underrepresented activities improved. For example, the *F*1 of the MLP’s ability to classify the *Open_Fridge* activity improved by more than 10 pp using the DBM, NDBM, and CBM.

**Figure 6:**
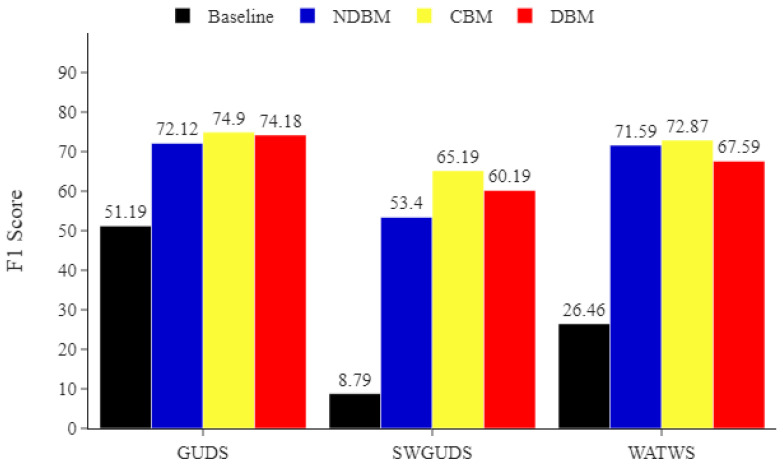
ADL minority classes. Comparing the impact of DBM, NDBM, and CBM on activity recognition performance, using MLP for the most underrepresented activities (*Going Up/Downstairs* (*GUDS*), *Standing Up, Walking and Going Up/Downstairs* (*SWGUDS*), and *Walking and Talking with Someone* (*WATWS*)). The reported means of *F*1 scores are obtained from 30 repetitions. The *F*1 score is in %.

On the ADL dataset, Figure 6 also suggests that by applying the DBM, NDBM, and CBM MLP classifier, *F*1 score was improved by more than 10 pp and gained a significant advantage in identifying the underrepresented activities, including *Going Up*/*Downstairs* (*GUDS*), *Standing Up*, *Walking and Going Up*/*Downstairs* (*SWGUDS*), and *Walking and Talking with Someone* (*WATWS*).

**Figure 7:**
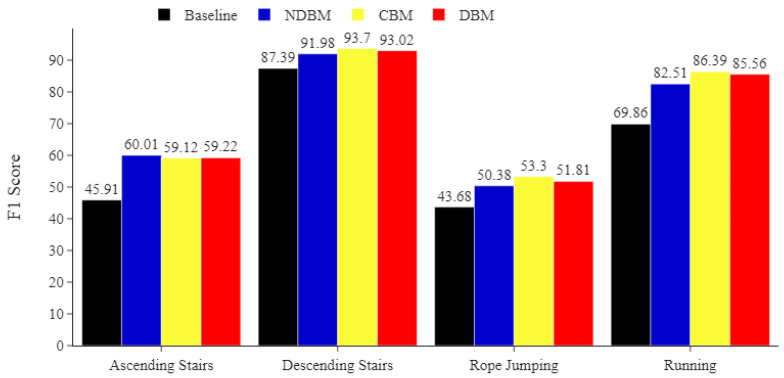
PAMAP2 minority classes. Comparing the impact of DBM, NDBM, and CBM on activity recognition performance, using MLP for the most underrepresented activities (ascending stairs, descending stairs, rope jumping, and running). The reported means of *F*1 scores are obtained from 30 repetitions. The *F*1 score is in %.

Similarly, on the PAMAP2 dataset, Figure 7 implies that the MLP classifier was more capable of identifying the underrepresented activities, including rope jumping, running, descending stairs, and ascending stairs, when the proposed DBM, NDBM, and CBM were used. For example, the performance of the MLP improved on the *F*1 score by at least 4 pp when identifying the underrepresented rope jumping activity.

### 6.6. Run Times for DBM, NDBM, and CBM

**Figure 8:**
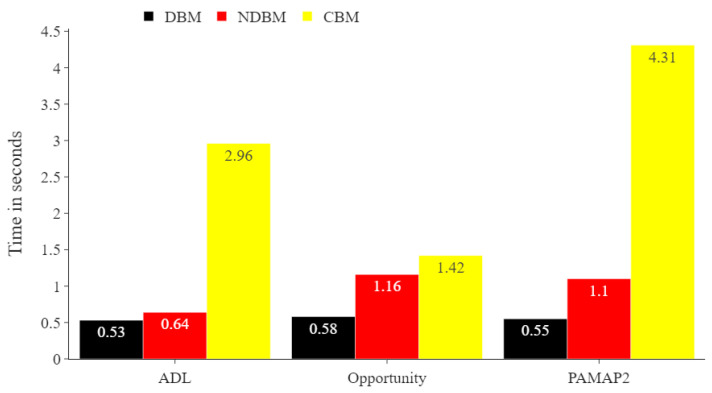
Comparing run times in seconds of the proposed DBM and CBM for all training datasets. The number of samples in the training sets for the ADL, Opportunity, and PAMAP2 datasets were 11,776, 1569, and 6450, respectively.

Figure 8 offers a comparison for each proposed sampling method in terms of run times. The analysis was performed on a Fierce PC with 16 GB RAM, Intel Core i7-7700 processor with 3.60 GHz and using Ubuntu 16.04 LTS (64-bits). DBM demonstrated the best performance in terms of training time compared to NDBM and CBM.

### 6.7. Statistical Analysis

A statistical analysis was performed to find out whether there are significant *F*1 performance differences between the nine sampling methods across five classifiers. The sampling methods analysed are SMOTE, Random_SMOTE, MSMOTE, SMOTE_TomeKLinks, CBSO, ProWSync, as well as the hybrid methods DBM, NDBM, and CBM. The classifiers are LR, RF, SVM, KNN, and MLP. The normality assumption is first estimated using the Anderson–Darling normality test on each sampling method and classifier combination [50,52]. This determines whether parametric statistical analysis, such as ANOVA, may be used in the case of normality, or a non-parametric method, such as the Friedman test, in the case of non-normality [53].

Table 8 shows the results of the Anderson–Darling normality test on sampling methods based on the five classifiers results for each dataset [52]. The mean *F*1 scores shown are obtained from 45 ’samples’, where one sample represents one sample method and classifier combination. On the PAMAP2 dataset, the Anderson–Darling *p*-value is more than 0.05 (α = 0.05)—suggesting the null hypothesis of a normal distribution—and so an ANOVA could then be used. For the ADL- and Opportunity-based results, Table 8 shows a rejection of the null hypothesis (*p* < 0.05) [52]. This indicates that these dataset results are not normally distributed and, therefore, ANOVA cannot be applied [54]. In its place, we use a Friedman test [55].

#### 6.7.1. ANOVA on PAMAP2

Table 9 reveals that the ANOVA test detected no statistical evidence to reject the null hypothesis (*p* > 0.05). In other words, when using PAMAP2, all sampling methods performed the same and none was found to perform significantly differently to the others.

#### 6.7.2. Friedman Test on ADL and Opportunity

The Friedman test in Table 10 indicates that the *p*-values of the data are less than 0.05 (α = 0.05) for the ADL and Opportunity datasets. Therefore, the null hypothesis is then violated. This means that there is a statistically significant difference across the sampling methods. In other words, one or more of the sampling methods can show different influences on these datasets.

Table 11 and Table 12 display the ranks drawn from the Friedman test in the ADL and Opportunity datasets [55]. The test compares rankings across the five classifiers (rows) and nine sampling methods (columns). Ranking is conducted for each classifier row, with sampling methods ranked between 1 (lowest) and 9 (high). The tables then summarize the total ranks obtained for each column to obtain the overall ranking for each sampling method [55].

Both Table 11 and Table 12 show that CBM has a consistently high ranking compared to the other sampling approaches across a range of classifiers. This supports our earlier finding that CBM is the highest performer.

## 7. Discussion and Future Work

Prior studies such as [2,4] have highlighted the lack of works that address and investigate the impact of the class imbalance problem in human activity recognition. Our present study fills this gap by proposing three approaches, DBM, NDBM, and CBM, to reduce the class imbalance and substantially improve human activity recognition (HAR) performance. We found that the proposed hybrid sampling methods worked better than applying any single sampling algorithm across three different HAR datasets. The benefit of the proposed approaches is that they generate more diverse samples, and thus improve the generalisability of the learning algorithm.

The cluster-based method (CBM) reveals consistently better performance than distance (DBM) or noise detection (NDBM)-based methods. A Friedman test additionally showed the statistical superiority of CBM over the other methods for two datasets, ADL and Opportunity, across five different classifiers.

Minority classes in particular benefit from using CBM, as shown in Figure 5, Figure 6 and Figure 7. This indicates that CBM would be a good choice when working with imbalanced HAR data involving activities similar to those found in Opportunity and ADL.

DBM, which is a combination of distance-based SMOTE and Random_SMOTE, provides the next highest performing combination. The main advantage of DBM, however, is that it uses significantly less computational resources than CBM. We suggest this method in instances where the training data suffer from small sample size and there is limited computational power. The main issue with DBM compared to NDBM and CBM is more likely to introduce noisy samples. The DBM does not perform any data filtering such as clustering processes prior to oversampling the data. One way to improve the DBM is to use a technique to assess the similarities between the synthetic samples and the training data samples (i.e., the original data), then to use only the most similar synthetic samples to the original sample in order to oversample the training data. For example, one can use the SMOTE and Random_SMOTE approaches to generate synthetic samples from the original training samples and use an efficient similarity metric such as Euclidean distance to compare the generated synthetic data to the original training samples and then use only the most similar synthetic samples and disregard the least similar. Our reason for this is that it might ensure that high-quality synthetic samples are used to oversample the training data.

Moreover, the key problem with the NDBM is that is relies on sampling methods that eliminate some samples during the oversampling process. This can lead to losing some valuable information of the activities. Therefore, we suggest that CBM be used by researchers to ensure they can be able to oversample the imbalance human activity data without losing any information.

To minimise complexity, we opted to use the default settings for most of the classifiers and sampling algorithms. Despite this, we believe that the general findings of the work regarding the influence of sampling on HAR still hold, and we have left further optimisation for future work.

One limitation of our proposed solutions is the choice of features. We chose to adopt time-domain features because these are efficient and fast to compute. This work might be extended by expanding on the feature set to incorporate, say, frequency domain features [12]. Additionally, we only considered data from a single accelerometer and a single location per dataset. How our sampling strategies might perform using an expanded feature set and a larger number of sensors will be the focus of future work.

A further area of future work will be to investigate more complex ensembles of sampling methods, e.g., combing distance with cluster-based methods. One challenge here will be to reduce the computational complexity of the clustering method, while preserving its ability to produce diverse samples.

## Figures and Tables

**Table 1:** Datasets details.

Dataset	Number of Subjects	Sample Rate	Window Size (s)	Sensor Position	Number of Sensors Used
Opportunity	4	32	2	Right Arm	1 accelerometer
PAMAP2	8	100	3	Dominant Wrist	1 accelerometer
ADL	15	52	10	Chest	1 accelerometer

**Table 2:** Features description [18,43].

Feature	Description
Mean	It provides the average value of sensor data within a segment
Standard deviation	It describes how much sensor data are spread around the mean
Minimum	The minimum value of sensor data within a segment
Maximum	The maximum value of sensor data within a segment
Median	It finds the middle number of a sample within a segment
Range	The difference between the maximum and the minimum of sensor data within a segment

**Table 3:** MLP architecture details.

Hidden Layers	Activation Function	Optimizer	Loss Function	Learning Rate	Regularization	Epochs
100	Relu	Adam	Cross-entropy	0.001	L2 penalty	200

**Table 4:** Distance-based method results. Comparing the performance of MLP on DBM, SMOTE, and Random_SMOTE for multiple datasets. The reported mean of *F*1 score and (±standard deviation), recall, and precision are obtained from 30 repetitions. The *F*1 score, recall, and precision are in %. Highest scores are shown in bold.

Data	Method	*F*1 Score	Recall	Precision
ADL	Baseline	87.2 (±0.047)	87.03	89.02
	SMOTE	92.24 (±0.069)	91.44	94.21
	Random_SMOTE	91.07 (±0.086)	90.31	93.22
	DBM	**92.59** (±0.081)	**91.9**	**94.26**
Opportunity	Baseline	28.85 (±0.017)	34.1	29.57
	SMOTE	42.95 (±0.043)	42.45	45.73
	Random_SMOTE	42.74 (±0.04)	42.19	45.75
	DBM	**48.49** (±0.052)	**48.18**	**50.63**
PAMAP2	Baseline	71.85 (±0.081)	72.73	75.49
	SMOTE	74.73 (±0.055)	74.93	77.69
	Random_SMOTE	74.59 (±0.055)	74.64	77.83
	DBM	**80.15** (±0.046)	**80.23**	**81.93**

**Table 5:** Noise detection-based results. Comparing the performance of MLP for NDBM, MSMOTE, and SMOTE_TomekLinks on multiple datasets. The reported mean of *F*1 score and (±standard deviation), recall, and precision are obtained from 30 repetitions. The *F*1 score, recall, and precision are in %. Highest scores are shown in bold.

Data	Method	*F*1 Score	Recall	Precision
ADL	Baseline	87.2 (±0.047)	87.03	89.02
	SMOTE_TomekLinks	91.41 (±0.071)	90.52	93.56
	MSMOTE	90.7 (±0.067)	89.65	92.66
	NDBM	**92.7** (±0.065)	**91.69**	**94.77**
Opportunity	Baseline	28.85 (±0.017)	34.1	29.57
	SMOTE_TomekLinks	42.89 (±0.039)	43.15	45.34
	MSMOTE	39.71 (±0.074)	39.58	42.07
	NDBM	**46.95** (±0.067)	**46.97**	**48.86**
PAMAP2	Baseline	71.85 (±0.081)	72.73	75.49
	SMOTE_TomekLinks	74.24 (±0.054)	74.51	77.13
	MSMOTE	73.73 (±0.059)	73.78	77.03
	NDBM	**79.43** (±0.054)	**79.46**	**81.35**

**Table 6:** Cluster-based results. Comparing the performance of MLP using CBM, CBSO, and ProWsyn on multiple datasets. The reported mean of *F*1 scores and (±standard deviation), recall, and precision are obtained from 30 repetitions. The *F*1 score, recall, and precision are in %. Highest scores are shown in bold.

Data	Method	*F*1 Score	Recall	Precision
ADL	Baseline	87.2 (±0.047)	87.03	89.02
	CBSO	91.16 (±0.09)	90.22	93.66
	ProWSyn	91.56 (±0.091)	90.98	93.7
	CBM	**92.96** (0.087)	**91.93**	**95.29**
Opportunity	Baseline	28.85 (±0.017)	34.1	29.57
	CBSO	42.92 (±0.023)	42.96	45.12
	ProWSyn	42.78 (±0.055)	43.47	44.99
	CBM	48.87 (±0.045)	**48.82**	**50.67**
PAMAP2	Baseline	71.85 (±0.081)	72.73	75.49
	CBSO	75.69 (±0.042)	75.43	78.19
	ProWSyn	74.42 (±0.054)	74.4	77.5
	CBM	**80.98** (±0.051)	**80.9**	**82.54**

**Table 7:** Comparing performance of DBM, NDBM, and CBM on multiple datasets. The reported mean of *F*1 scores and (±standard deviation), recall, and precision were obtained from 30 repetitions. The *F*1 score, recall, and precision are in %. Highest scores are shown in bold.

Data	Method	*F*1 Score	Recall	Precision
ADL	Baseline	87.2 (±0.047)	87.03	89.02
	DBM	92.59 (±0.081)	91.9	94.26
	NDBM	92.7 (±0.065)	91.69	94.77
	CBM	**92.96** (±0.087)	**91.93**	**95.29**
Opportunity	Baseline	28.85 (±0.017)	34.1	29.57
	DBM	48.49 (±0.052)	48.18	50.63
	NDBM	46.95 (±0.067)	46.97	48.86
	CBM	**48.87** (±0.045)	**48.82**	**50.67**
PAMAP2	Baseline	71.85 (±0.081)	72.73	75.49
	DBM	80.15 (±0.046)	80.23	81.93
	NDBM	79.43 (±0.054)	79.46	81.35
	CBM	**80.98** (±0.051)	**80.9**	**82.54**

**Table 8:** Anderson–Darling normality test on sampling methods based on the 5 classifiers results × 9 sampling methods (5 × 9 = 45 sample size) on each dataset. The *p*-value is less than 0.05 (α = 0.05) for ADL and Opportunity which suggests that ADL and Opportunity are not normally distributed compared to PAMPA2.

Data	Mean	Standard Deviation	Sample Size	*p*-Value
ADL	0.8840	0.0399	45	0.0007
Opportunity	0.3773	0.0548	45	0.0000
PAMAP2	0.7272	0.0406	45	0.0680

**Table 9:** ANOVA for PAMAP2 dataset.

Data	Degrees of Freedom	Sum of Squares	Mean Square	F Value	*p*-Value
PAMAP2	8	0.0067	0.0008	0.4602	0.8757

**Table 10:** Friedman test results indicate that the *p*-value is less than 0.05 (α = 0.05) for the ADL and Opportunity datasets. This means that one or more of the sampling methods is more effective than the others.

Data	Degrees of Freedom	Chi-Square	*p*-Value
ADL	8	21.8133	0.0053
Opportunity	8	24.2133	0.0021

**Table 11:** Friedman sum-of-ranks test on ADL-based results for all methods and classifiers. CBM is the overall highest ranking method.

Classifier	CBSO	NDBM	CBM	DBM	MSMOTE	Pro-WSyn	Random_SMOTE	SMOTE_TomekLinks	SMOTE
KNN	1	7	9	4	5	8	2	6	3
LR	1	8	3	9	2	5	6	7	4
MLP	3	8	9	7	1	5	2	4	6
RF	1	6	9	4	7	8	3	5	2
SVM	1	8	7	9	2	3	6	4	5
Sum of ranks	7	37	**37**	33	17	29	19	26	20

**Table 12:** Friedman sum-of-ranks test on Opportunity-based results for all methods and classifiers. CBM is the overall highest ranking method.

Classifier	CBSO	NDBM	CBM	DBM	MSMOTE	Pro-WSyn	Random_SMOTE	SMOTE_TomekLinks	SMOTE
KNN	5	6	9	7	1	4	8	3	2
LR	5	9	7	8	1	2	6	4	3
MLP	5	7	9	8	1	3	2	4	6
RF	4	5	8	3	1	9	7	6	2
SVM	2	7	8	9	1	4	3	5	6
Sum of ranks	21	34	**41**	35	5	22	26	22	19

**Figure A1:**
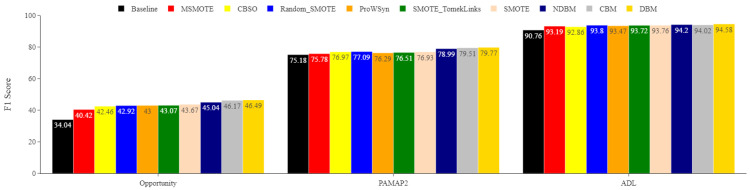
*F*1 score of baseline (SVM), the proposed method, and the sampling methods for the Opportunity, PAMAP2, and ADL datasets. The reported means of *F*1 scores were obtained from 30 repetitions.

**Figure A2:**
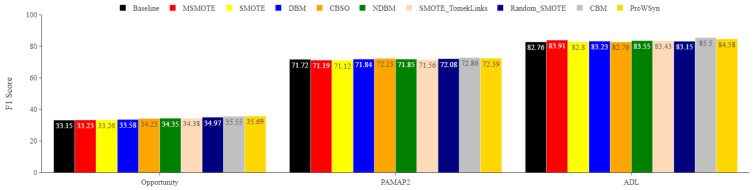
*F*1 score of baseline (RF), the proposed method, and the sampling methods for the Opportunity, PAMAP2, and ADL datasets. The reported means of *F*1 scores were obtained from 30 repetitions.

**Figure A3:**
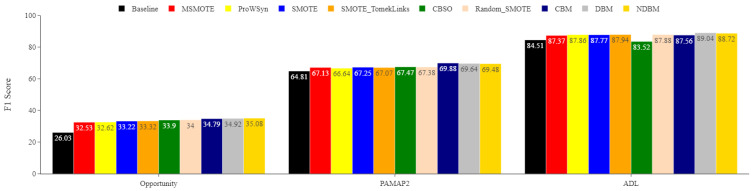
*F*1 score of baseline (LR), the proposed method, and the sampling methods for the Opportunity, PAMAP2 and ADL datasets. The reported means of *F*1 scores were obtained from 30 repetitions.

**Figure A4:**
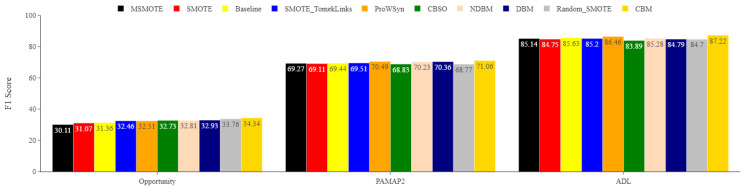
*F*1 score of baseline (KNN), the proposed method, and the sampling methods for the Opportunity, PAMAP2, and ADL datasets. The reported means of *F*1 scores were obtained from 30 repetitions.

## Appendix A

**Table A1:** Parammeters settting of SVM, LR and KNN on multiple dataset. Ref. [47] provides description about the parameters that we used.

Algorithms	Parameters	ADL	Opportunity	PAMAP2
SVM	gamma	0.1	0.1	0.1
	C	20	20	20
	kernel	rbf	rbf	rbf
	max_iter	−1	−1	−1
	decision_function_shape	ovr	ovr	ovr
LR	multi_class	multinomial	multinomial	multinomial
	solver	newton-cg	sag	sag
	max_iter	250	250	250
	C	2	2	2
	penalty	L2	L2	L2
KNN	n_neighbors	3	5	3
	algorithm	auto	auto	auto

## Appendix B

**Table A2:** Comparing the performance of the baseline classifiers on multiple datasets. The reported mean of *F*1 scores and (±standard deviation) were obtained from 30 repetitions. The *F*1 score is as %.

Data	Classifier	*F*1 Score
ADL	KNN	85.63 (±0.043)
	LR	84.51 (±0.026)
	MLP	87.2 (±0.047)
	RF	82.76 (±0.037)
	SVM	90.76 (±0.037)
Opportunity	KNN	31.36 (±0.052)
	LR	26.03 (±0.012)
	MLP	28.85 (±0.017)
	RF	33.15 (±0.032)
	SVM	34.04 (±0.012)
PAMAP2	KNN	69.44 (±0.033)
	LR	64.81 (±0.094)
	MLP	71.85 (±0.081)
	RF	71.72 (±0.057)
	SVM	75.18 (±0.06)

## Appendix C

We exhibited the *F*1 score of the baseline classifiers including the SVM, RF, LR and KNN in order to compare the influence of the sampling methods in improving their *F*1 score. The sampling methods were the proposed DBM, NDBM and CBM as well as the existing methods including, SMOTE, Random_SMOTE, SMOTE_Tomeklinks, MSMOTE, CBSO, and ProWSyn.

The below figures compared the *F*1 scores of the SVM, RF, LR, and KNN on the Opportunity, PAMAP2, and ADL datasets. For more details about the dataset, see Section 4.
